# Effect of* Moringa oleifera* Leaf Capsules on Glycemic Control in Therapy-Naïve Type 2 Diabetes Patients: A Randomized Placebo Controlled Study

**DOI:** 10.1155/2017/6581390

**Published:** 2017-11-28

**Authors:** Rutchaporn Taweerutchana, Natchagorn Lumlerdkij, Sathit Vannasaeng, Pravit Akarasereenont, Apiradee Sriwijitkamol

**Affiliations:** ^1^Division of Endocrinology and Metabolism, Department of Medicine, Faculty of Medicine Siriraj Hospital, Mahidol University, Salaya, Thailand; ^2^Center of Applied Thai Traditional Medicine, Faculty of Medicine Siriraj Hospital, Mahidol University, Salaya, Thailand; ^3^Department of Pharmacology, Faculty of Medicine Siriraj Hospital, Mahidol University, Salaya, Thailand

## Abstract

**Background:**

Studies showed effects of* Moringa oleifera* (MO) on lowering blood sugar levels in animal and diabetes patients. The aims of this study were to determine the effect of MO leaf capsules on glucose control in therapy-naïve type 2 diabetes mellitus (T2DM) and to evaluate its safety.

**Method:**

This was a prospective randomized placebo controlled study. Therapy-naïve T2DM was randomly assigned to receive either 8 grams per day of MO leaf capsules (MO leaf group) or placebo for 4 weeks. Clinical and laboratory characteristics were recorded at screening and at the end of 4-week study. 9-point plasma glucose was obtained before and every week during the study.

**Results:**

Thirty-two T2DM patients were enrolled. The mean age was 55 years and the mean HbA1C was 7.0%. There was no significant difference in FPG and HbA1C between groups. MO leaf group had SBP reduction by 5 mmHg as compared to baseline but this difference had no statistical significance. There were no adverse effects of MO leaf.

**Conclusions:**

* Moringa oleifera* leaf had no effect on glycemic control and no adverse effects in T2DM. Interestingly, this study demonstrated that MO leaf had a tendency on blood pressure reduction in T2DM, and this result needs further investigation.

## 1. Introduction

Type 2 diabetes mellitus (T2DM) is a major public health problem. Insulin resistance and impairment of pancreatic insulin secretion are the main pathogenesis of T2DM. Treatment of T2DM and its complications is usually complicated and costly. Herbal medicines have long been used as an alternative treatment for type 2 diabetes.* Moringa oleifera *(MO) drumstick tree is a traditional herb widely used for a long time. In Thailand, we use MO seed as an important ingredient in Thai-traditional food. In Western Asia, most parts of the plant are well known for their pharmacological actions and have been used as antihypertensive drugs, thyroid hormone regulator, laxatives, and antibiotics [1–3]. Several studies [4–6] in nondiabetic and diabetic rat model have shown that MO leaf could decrease plasma and urine glucose and improve glucose tolerance test. These hypoglycemic effects were postulated to be associated with decrease intestinal glucose uptake and slowing gastric emptying time by fiber in MO leaf. Our previous study [7] conducted in 10 healthy volunteer demonstrated that a single dose of 4 grams of MO leaf powder capsules significantly increased insulin secretion in healthy subjects without any adverse effects on liver and kidney function. Despite several evidence of benefit of* Moringa oleifera* on plasma glucose in anima model and healthy subjects, the data in type 2 diabetes are still lacking. There are a few studies [8, 9] which evaluated an efficacy and safety of* Moringa oleifera* in type 2 diabetes patients; however those studies are not randomized; therefore, this study was conducted to determine whether the use of the MO leaf capsules would improve glucose control in patients with therapy-naïve type 2 diabetes mellitus (T2DM) in a randomized placebo controlled study and to evaluate its safety.

## 2. Materials and Methods

This study was a prospective randomized placebo controlled study conducted from July 2012 to February 2013 at Siriraj Hospital, Faculty of Medicine Siriraj Hospital, Mahidol University, Thailand. Siriraj Institutional Review Board approved the study and all subjects gave written consent.

### 2.1. Participants

Participants were eligible to enroll in the study if they were therapy-naïve type 2 diabetes with the duration of diabetes of less than 5 years, age between 20 and 70 years, hemoglobin A1C (HbA1C) of less than 9%, and fasting plasma glucose of less than 200 mg/dl. Exclusion criteria were type 1 or others types of diabetes, use of glycemic lowering agents within 2 months before the enrollment, a creatinine clearance of less than 60 ml/min./1.73 m^2^, an elevation of alanine aminotransferase or aspartate aminotransferase level of more than 2 times of the upper limit of the normal range, and history of heart disease or other serious illness and pregnancy.

### 2.2. Calculation of Sample Size

The sample size was calculated by

(1) $$\begin{array}{c} n\;\;per\;\;group=\frac{2{\left\{\left(Z_{\alpha /2}+Z_{\beta }\right)SD\right\}}^{2}}{d^{2}}. \end{array}$$

Definition is as follows:

 
*α* = type I error (2-sided test) = 0.05; *Z*_0.025_ = 1.96.
 
1 − *β* = Power = 0.80; *Z*_0.2_ = 0.84.
  SD = SD of HbA_1c_ = 0.95 (reference value of our institute).
 
*d* = the clinical significance of Hb_A1c_ between each group was 1 *n* and therefore per group was 16 persons.

The follow-up period of this study was 1 month with the expected dropout being 10%; therefore, the number of participants in each group should be 18 persons.

### 2.3. Study Procedure

**Figure 1:**
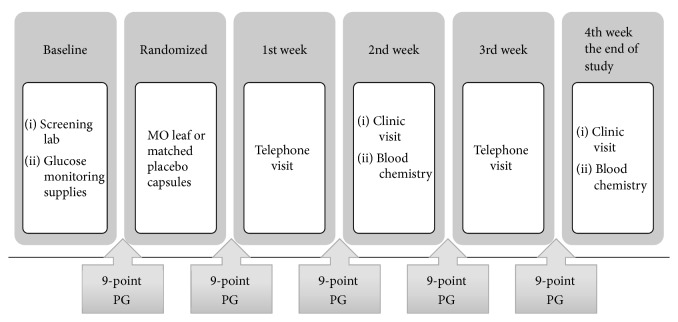
Study procedures.

The study consisted of a screening period (1-2 weeks) and a randomized treatment period (4 weeks). At the screening period, all participants came to the diabetes unit for history taking, physical examination including body weight and blood pressure and laboratory testing including FPG, HbA1C, creatinine, and liver function test. They also received behavioral counseling regarding diabetes care and learned how to do the 9-point plasma glucose (PG) monitoring, using ACCU-CHECK Advantage meter system (Roche Thailand). The 9-point plasma glucose (PG) profile was obtained in each participant during the study period as shown in Figure 1. The 9-point PG profile included PG measurement in the morning of day 1, PG measurement before each meal (premeal PG), 2 hours after each meal (postmeal PG) and at bedtime of day 2, and PG measurement before breakfast of day 3. If the participants did not meet the exclusion criteria, they were randomized, using table of random numbers assigned in a 1 : 1 ratio, to receive either* Moringa oleifera* leaf capsule or matched placebo. During 4-week treatment period, all participants randomly received either 8 capsules (4 grams) of MO leaf capsule or matched placebo, before breakfast and dinner. The dosage of MO leaf used in this study was based on our previous study that demonstrated the effect of MO leaf capsule on insulin secretion in healthy subjects [7]. At the end of 4-week study, all the participants were asked to come back to diabetes unit for history taking, physical examination, and blood drawing for measurement of FPG, HbA1C, creatinine, and liver function test.

### 2.4. Definitions

Plasma glucose data obtained from 9 point PG profile were used to calculate mean daily PG, mean premeal PG, and mean postmeal PG.

- *Mean daily PG* was defined as the average of 9 point PG in each visit.
- *Mean premeal PG *was calculated using PG of day 1, premeal PG from each meal of day 2, and PG of day 3.
- *Mean postmeal PG* was using postmeal PG from each meal of day 2.

Hypoglycemia was diagnosed by either PG measurement of less than 70 mg/dl or patient experienced episodes of low blood sugar with or without PG confirmed.

### 2.5. Study Drugs

#### 2.5.1. *Moringa oleifera* Leaf Capsules

A single batch of* Moringa oleifera* leaf powder capsules was manufactured at the Herbal Medicine and Products Manufacturing unit, Center of Applied Thai Traditional Medicine, Faculty of Medicine Siriraj Hospital, Mahidol University (Bangkok, Thailand), according to good manufacturing practice guidelines. The dried leaves of* Moringa oleifera* were separated from foreign matters, cleaned, and oven-dried. Then they were ground and sifted to be powder. The powder was filled into capsule shells, and each capsule contains 500 mg of dried leaf powder of* Moringa oleifera*.

#### 2.5.2. Placebo Capsules

Placebo capsules were also manufactured at the Herbal Medicine and Products Manufacturing. Each placebo capsules had size and color identical to* Moringa oleifera* leaf capsules and consisted of plain powder, magnesium stearate, and talcum.

## 3. Statistical Analyses

The SPSS version 18.0 was used for all analyses. All continuous variables were expressed as mean ± SD or median (min, max) as appropriated. The categorical variables were shown as frequency and percentage. The independent* t*-test was used for the continuous variables and Chi-square and Fisher test were used for the categorical variables. The differences of glucose parameter between the two treatment arms and the change between baseline and endpoint were determined using the repeated measured ANOVA. Due to small sample size, *P* for trend was used to analyze the linear trend of one-way ANOVA. For all analyses, *P* < 0.05 was considered to be statistically significant.

## 4. Results

### 4.1. Subject Characteristic

A total of 32 therapy-naïve type 2 diabetes patients were enrolled in the study. All participants completed the study with the compliance of the study drugs greater than 80%. The 9 point PG profiles were completed with the missing data only 2%.  Table 1 summarized the subject's clinical and laboratory characteristics. There were no differences in age, sex, the duration of T2DM, BW, BMI, FPG, and HbA1C between two groups.

### 4.2. Effect of MO Leaf Capsule in Therapy-Naïve T2DM

At the end of study, there was a nonsignificant decrease in body weight by 1 kg in both groups. Interestingly, despite no change in antihypertensive agents, MO leaf group had a reduction of SBP and DBP by 5 mmHg as compared to baseline, whereas placebo group had an increase in blood pressure by 2 mmHg as compared to baseline; however these differences had no statistical significance (Table 2).

**Figure 2:**
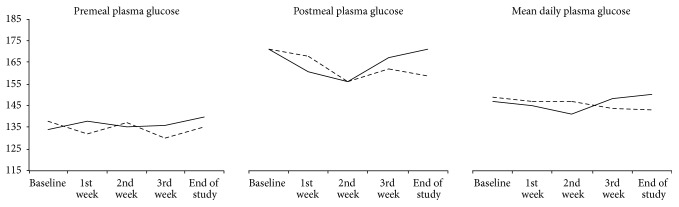
Mean premeal, postmeal, and daily plasma glucose in MO leaf and placebo group. The solid line and dash line represented MO leaf and placebo group, respectively.

There was no significant difference of FPG and HbA1C between MO leaf and placebo group. Four weeks of treatment caused 0.2–0.3% reduction of HbA1C as compared to baseline in both treatment arms (Table 2) but these changes did not reach statistical significance. The mean daily PG, mean premeal, and mean postmeal PG in MO leaf group were not different from those in placebo group (Figure 2).

### 4.3. Safety of MO Leaf Capsule in Therapy-Naïve T2DM

There was no incidence of hypoglycemia in both treatment groups. Four of 16 patients (25%) in MO group reported transient diarrhea, which is spontaneously resolved within a few days. There were no differences in BUN, Cr, AST, and ALT between baseline and the end of study in both groups. These data demonstrated that there were no adverse effects after high dose of MO leaf powder.

## 5. Discussion

This study is the first randomized placebo controlled clinical study that compared the effect of* Moringa oleifera* leaf capsules and placebo in therapy-naïve type 2 diabetes patients. In this study, we found tend toward a decrement in hemoglobin A1C in both MO leaf and placebo group, but these changes did not reach statistical significance. There was no difference in fasting, premeal, and postmeal plasma glucose between two treatment arms. There was no adverse effect of the use of MO leaf. Interestingly, we found a decrease in systolic and diastolic blood pressure in MO leaf group; however the change did not reach statistical significance.

Previous studies using* Moringa oleifera* leaf demonstrated the glucose lowering effect of MO leaf in animal and human studies [8, 9]; however this study did not demonstrated the similar effect. Our previous study [7] in healthy volunteer has shown that a single dose of 4 grams of MO leaf powder capsules significantly increased insulin secretion by 74%; therefore the lack of effect on plasma glucose in this study should not be explained by an inappropriate dosage.

The discrepancy between this study and others might be explained by several reasons. First of all, it is because a short period of this study; therefore it was too early to demonstrate the change in HbA1C that need at least 8–12 weeks. However, we did not observe any change in fasting, premeal, or postmeal plasma glucose, which could change earlier, as had been demonstrated in previous 2-week study [9]. Second, the hypoglycemic effects of MO leaf have been postulated to be associated with decreased intestinal glucose uptake and slowing gastric emptying time by fiber in MO leaf, which contained fiber 12% (w/w) [10] and had an effect on postprandial plasma glucose by three important bioactive phytochemicals including quercetin, chlorogenic acid, and moringinine [11, 12]. Quercetin, a potent antioxidant, showed antidiabetic effects in Zucker rat, the insulin resistance model [13]. Chlorogenic acid has been shown to inhibit glucose-6-phosphate translocase in rat liver which resulted in a reduction of hepatic gluconeogenesis and glycogenolysis [14, 15]. In human study, chlorogenic acid showed a decrease in glycemic response during oral glucose tolerance test [16]. Moringinine demonstrated an improvement of glucose tolerance in rat model [17]. We used the raw material in this study which might contained only a small amount of these bioactive phytochemicals that could explain why we could not demonstrated the effect on postmeal glucose. Lastly, the improvement of plasma glucose in placebo group might be an effect of self-monitoring of blood glucose (SMBG), so we could not demonstrate the difference between two treatment arms. Data from Cochrane review in 2012 [18] showed that a short-term follow-up, up to six months in newly diagnosed type 2 diabetes who had SMBG, had a statistically significant decrease of HbA1C by 0.3% (95% CI −0.4 to −0.1) as compared with the control group. On the other hand, in a long-term study, over a 12-month follow-up period, this effect was diminished, which resulted in a decrement of HbA1C by 0.1% in SMBG groups as compared to control. The effect of SMBG on glycemic control could be explained by its feedback on patient behavior to empower the patient to gain control over their disease and to motivate the lifestyle changes [19, 20]. Therefore, longer study period should be done to eliminate an effect of SMBG on glycemic control, and thus we could distinguish the effect of MO leaf as compared to placebo.

The second aim of this study was to evaluate the safety of* Moringa oleifera* leaf. It has been shown in animal studies that the high dose of MO leaf resulted in transaminitis, followed by weight gain [21]. However, we found no adverse effects on liver and kidney function in our subjects. Twenty-five percent of patients who took MO leaf powder reported short duration of diarrhea with resolved spontaneously. Previous human studies of MO leaf also reported no adverse event similar to this study [8, 22, 23].

We also found a trend toward blood pressure reduction after MO leaf ingestion. Recent meta-analysis found that every 5 mmHg reduction of systolic blood pressure resulted in significant reduction of cardiovascular disease and all-cause mortality [24]; therefore this blood pressure lowering effect would have clinical benefits in diabetes patients. The antihypertensive effect of MO leaves had been shown in previous review [2]. Recent animal studies [25] have shown that MO leaf exerts antihypertensive effects by inhibiting the secretion of IL-2 and modulates T-cell calcium signaling in hypertensive rats. Therefore, further study of blood pressure lowering effect of* Moringa oleifera *is needed.

The limitation of this study was a short duration of the study so the larger and longer duration of study are needed before we can draw a conclusion about the effect of MO leaf on plasma glucose.

In conclusion,* Moringa oleifera* leaf had no effect on glycemic control in T2DM in this short-term study; however, we could demonstrate that the use of MO did not have any adverse effects. Interestingly, this study demonstrated that MO leaf had a tendency on blood pressure reduction in T2DM patients; this result needs further investigation.

## Figures and Tables

**Table 1:** Baseline clinical and laboratory characteristics of MO leaf and placebo group.

	MO leaf (*n* = 16)	Placebo (*n* = 16)	*P* value
Male sex	7 (44%)	10 (56%)	0.48
Age (years)	52 ± 11	57 ± 7	0.07
Body weight (kg)	73 ± 12	70 ± 12	0.91
BMI (kg/m^2^)	28.1 ± 4.6	27.1 ± 3.2	0.14
Duration of DM (months)	18 (1–48)	18 (2–60)	0.72
Family history of DM	14 (88%)	12 (75%)	0.65
Hypertension	7 (44%)	10 (63%)	0.29
Antihypertensive agents (%)	7 (22%)	9 (28%)	1.00
Dyslipidemia	9 (56%)	13 (81%)	0.13
Lipid lowering agents (%)	5 (16%)	11 (34%)	0.28
FPG (mg/dl)	138 ± 35	132 ± 28	0.14
HbA1C (%)	7.1 ± 0.9	6.9 ± 0.7	0.39

MO leaf, *Moringa oleifera* leaf; BMI, body mass index; DM, diabetes mellitus; FPG, fasting plasma glucose; HbA1C, hemoglobin A1C.

**Table 2:** Clinical and glucose parameter in MO leaf and placebo group.

	MO leaf	Placebo	*P* value^A^	*P* value^B^
Body weight (kg)				
(i) Baseline	73 ± 12	70 ± 12	0.49	
(ii) 2nd week	73 ± 12	70 ± 12	0.51	
(iii) End of study	72 ± 12	69 ± 12	0.52	0.12
SBP (mmHg)				
(i) Baseline	133 ± 16	128 ± 14	0.36	
(ii) 2nd week	133 ± 13	126 ± 15	0.14	
(iii) End of study	128 ± 13	130 ± 17	0.68	0.25
DBP (mmHg)				
(i) Baseline	85 ± 11	76 ± 9	0.03	
(ii) 2nd week	82 ± 8	76 ± 9	0.11	
(iii) End of study	79 ± 8	79 ± 12	0.94	0.17
FPG (mg/dl)				
(i) Baseline	138 ± 36	132 ± 28	0.14	
(ii) 2nd week	130 ± 30	129 ± 25	0.33	
(iii) End of study	136 ± 34	126 ± 29	0.44	1.00
HbA1C (%)				
(i) Baseline	7.14 ± 0.97	7.15 ± 1.01	0.49	
(ii) End of study	6.93 ± 0.73	6.87 ± 0.72	0.37	0.82

MO leaf, *Moringa oleifera* leaf; SBP, systolic blood pressure; DBP, diastolic blood pressure; FPG, fasting plasma glucose; HbA1C, hemoglobin A1C; PG, plasma glucose; ^A^*P* value compare between MO leaf and placebo; ^B^*P* value compare between baseline and end of study.

## References

[1] Fahey J. W. (2005). Moringa oleifera: a review of the medical evidence for its nutritional, therapeutic, and prophylactic properties. part 1. *Trees for Life Journal*.

[2] Anwar F., Latif S., Ashraf M., Gilani A. H. (2007). *Moringa oleifera*: a food plant with multiple medicinal uses. *Phytotherapy Research*.

[3] Thurber M. D., Fahey J. W. (2009). Adoption of Moringa oleifera to combat under-nutrition viewed through the lens of the "Diffusion of innovations" theory. *Ecology of Food and Nutrition*.

[4] Makonnen E., Hunde A., Damecha G. (1997). Hypoglycaemic effect of Moringa stenopetala aqueous extract in rabbits. *Phytotherapy Research*.

[5] Kar A., Choudhary B. K., Bandyopadhyay N. G. (2003). Comparative evaluation of hypoglycaemic activity of some Indian medicinal plants in alloxan diabetic rats. *Journal of Ethnopharmacology*.

[6] Ndong M., Uehara M., Katsumata S.-I., Suzuki K. (2007). Effects of oral administration of *Moringa oleifera* Lam on glucose tolerance in Goto-Kakizaki and wistar rats. *Journal of Clinical Biochemistry and Nutrition*.

[7] Anthanont P., Lumlerdkij N., Akarasereenont P., Vannasaeng S., Sriwijitkamol A. (2016). Moringa oleifera leaf increases insulin secretion after single dose administration: A preliminary study in healthy subjects. *Journal of the Medical Association of Thailand*.

[8] William F., Lakshminarayanan S., Chegu H. (1993). Effect of some indian vegetables on the glucose and insulin response in diabetic subjects. *International Journal of Food Sciences and Nutrition*.

[9] John S., Chellappa A. R. (2005). Hypoglycemic effect Oleifera powder diabetic subjects rats. *Indian Journal of Nutrition and Dietetics*.

[10] Joshi P., Mehta D. (2010). Effect of dehydration on nutritive value of drumstick leaves. *Journal of Metabolomics and Systems Biology*.

[11] Bennett R. N., Mellon F. A., Foidl N. (2003). Profiling glucosinolates and phenolics in vegetative and reproductive tissues of the multi-purpose trees *Moringa oleifera* L. (Horseradish tree) and *Moringa stenopetala* L. *Journal of Agricultural and Food Chemistry*.

[12] Manguro L. O. A., Lemmen P. (2007). Phenolics of Moringa oleifera leaves. *Natural Product Research (Formerly Natural Product Letters)*.

[13] Rivera L., Morón R., Sánchez M., Zarzuelo A., Galisteo M. (2008). Quercetin ameliorates metabolic syndrome and improves the inflammatory status in obese Zucker rats. *Obesity*.

[14] Karthikesan K., Pari L., Menon V. P. (2010). Antihyperlipidemic effect of chlorogenic acid and tetrahydrocurcumin in rats subjected to diabetogenic agents. *Chemico-Biological Interactions*.

[15] Karthikesan K., Pari L., Menon V. P. (2010). Combined treatment of tetrahydrocurcumin and chlorogenic acid exerts potential antihyperglycemic effect on streptozotocin-nicotinamide-induced diabetic rats. *General Physiology and Biophysics*.

[16] Tunnicliffe J. M., Eller L. K., Reimer R. A., Hittel D. S., Shearer J. (2011). Chlorogenic acid differentially affects postprandial glucose and glucose-dependent insulinotropic polypeptide response in rats. *Applied Physiology, Nutrition, and Metabolism*.

[17] Bour S., Visentin V., Prévot D. (2005). Effects of oral administration of benzylamine on glucose tolerance and lipid metabolism in rats. *Journal of Physiology and Biochemistry*.

[18] Malanda U. L., Welschen L. M. C., Riphagen I. I., Dekker J. M., Nijpels G., Bot S. D. M. (2012). Self-monitoring of blood glucose in patients with type 2 diabetes mellitus who are not using insulin.. *Cochrane Database of Systematic Reviews (Online)*.

[19] Farmer A., Wade A., Goyder E. (2007). Impact of self monitoring of blood glucose in the management of patients with non-insulin treated diabetes: Open parallel group randomised trial. *British Medical Journal*.

[20] Farmer A. J., Wade A. N., French D. P. (2009). Blood glucose self-monitoring in type 2 diabetes: A randomised controlled trial. *Health Technology Assessment*.

[21] Adedapo A. A., Mogbojuri O. M., Emikpe B. O. (2009). Safety evaluations of the aqueous extract of the leaves of *Moringa oleifera* in rats. *Journal of Medicinal Plants Research*.

[22] Kumari D. J. (2010). Hypoglycaemic effect of Moringa oleifera and Azadirachta indica in type 2 diabetes mellitus. *Bioscan*.

[23] Giridhari V. V. A., Malathi D., Geetha K. (2011). Anti diabetic property of drumstick (Moringa oleifera) leaf Tablets. *International Journal of Health and Nutrition*.

[24] Bundy J. D., Li C., Stuchlik P. (2017). Systolic Blood Pressure Reduction and Risk of Cardiovascular Disease and Mortality. *JAMA Cardiology*.

[25] Attakpa E. S., Chabi N. W., Bertin G. A., Ategbo J. M., Seri B., Khan N. A. Moringa oleifera-rich diet and T-cell calcium signaling in hypertensive rats. *Physiological Research*.

